# The efficacy and safety of tranexamic acid in high tibial osteotomy: a systematic review and meta-analysis

**DOI:** 10.1186/s13018-021-02512-4

**Published:** 2021-06-11

**Authors:** Jimin Ma, Hanli Lu, Xinxing Chen, Dasai Wang, Qiang Wang

**Affiliations:** Department of Orthopedics, Yijishan Hospital of Wannan Medical College, Wuhu, Anhui China

**Keywords:** Blood loss, Hemoglobin decrease, Drain output, High tibial osteotomy, Tranexamic acid, Meta-analysis

## Abstract

**Objective:**

The present meta-analysis was conducted to evaluate the efficacy and safety of the application of tranexamic acid (TXA) in patients undergoing high tibial osteotomy (HTO).

**Methods:**

PubMed (MEDLINE), EMBASE, and Cochrane Library were systematically searched for relevant literature from inception until 1 February 2021. A combined searching strategy of subject words and random words was adopted. After testing for potential publication bias and/or heterogeneity, we aggregated variables by using the random-effect model. The primary comparison outcome measures were total blood loss, hemoglobin decrease, drain output, wound complications, thrombotic events, and blood transfusion rate of the TXA group versus control. The meta-analysis was performed using the RevMan 5.3 software.

**Results:**

A total of 5 studies were included involving 532 patients. The results showed that there were significant differences in the two groups concerning total blood loss (95% confidence interval [CI] − 332.74 to − 146.46, *P* < 0.00001), hemoglobin decrease on postoperative day (POD) 1, 2, and 5 (POD 1 95% CI − 1.34 to − 0.63, *P* < 0.00001; POD 2 95% CI − 1.07 to − 0.68, *P* < 0.00001; POD 5 95% CI − 1.46 to − 0.84, P < 0.00001), drain output (POD total 95% CI − 195.86 to − 69.41, *P* < 0.00001) and wound complications (RR = 0.34, 95% CI 0.12 to 0.97, *P* = 0.04). Nonsignificant differences were found in the incidence of thromboembolic events (RR = 0.46, 95% CI 0.09 to 2.41, *P* = 0.36) and blood transfusion rate (RR = 0.25, 95% CI 0.03 to 2.27, *P* = 0.22).

**Conclusions:**

This meta-analysis of the available evidence demonstrated that TXA could reduce total blood loss, hemoglobin decrease, drain output, and wound complications without increasing the incidence of thromboembolic events in patients undergoing HTO. But there is no obvious evidence that TXA could reduce blood transfusion rates. Further studies, including more large-scale and well-designed randomized controlled trials, are warranted to assess the efficacy and safety issues of routine TXA use in HTO patients.

## Introduction

High tibial osteotomy (HTO) is a widely used and well-established effective surgical treatment which aims to correct the varus malalignment or deformation with compartmental osteoarthritis or osteonecrosis of the knee in both young and elderly patients [1]. HTO is intended to shift the misaligned mechanical axis to the midline of the knee to restore normal limb alignment, thereby minimizing the load on the medial compartment to slow down the osteoarthritis progression [2–4]. Furthermore, HTO can also be implemented to delay the urgent need for a total or partial knee replacement procedure by preserving the damaged tissue in the knee joint. However, this surgical procedure can cause extensive bleeding due to bone gap and release of blood vessels as well as extensive soft tissues in and around the damaged site [5, 6], that may lead to soft tissue complications, such as wound hematoma, superficial skin infections, delayed union, and even compartment syndrome [7–9].

Tranexamic acid (TXA) is routinely used to stop heavy bleeding and to reduce the transfusion requirements in patients during peri- and post-operative periods. Due to its anti-fibrinolytic property, it is commonly used in total hip and knee arthroplasty [10–14]. TXA is a synthetic lysine analog that competitively blocks the lysine binding site on fibrinolysin and plasminogen, thus inhibiting the activation of plasminogen to plasmin, and also promotes clot formation by regulating the platelet dispersions [15, 16].

Orthopedic surgeons play critical roles in bridging the gaps between basic science research and its practical clinical applications by integrating recent transformative discoveries into real-life orthopedic practice [17, 18]. The mechanistic roles of TXA in reducing total blood loss and blood transfusion events are well documented in many surgical specialties, including orthopedics, cardiology, neurosurgery, craniofacial surgery, and obstetrics, and gynecology [14]. Furthermore, the application of TXA has not been shown to increase the risks of thromboembolic events in these cases. However, the clinical value of TXA has not been extensively evaluated in patients undergoing HTO. In part because many surgeons or medical practitioners are still not aware of the higher efficacy and safety of TXA over other routinely used drugs in HTO. To overcome this bottleneck, we performed this meta-analysis to systematically review the efficiency of TXA to control total blood loss, drain output, hemoglobin-decrease, and wound complications.

## Methods

This meta-analysis was performed according to the Preferred Reporting Items for Systematic Reviews and Meta-Analyses (PRISMA) guidelines. Ethics approval and participants’ consent were not required for this study, as it is based on previously published study reports.

### Literature search

PubMed (MEDLINE), EMBASE, and Cochrane Library were comprehensively searched by 2 independent reviewers from inception until 1 February 2021. A combined searching strategy of subject words and random words was used: (((("tibia"[Mesh]) OR tibia) OR high) OR proximal) OR (("osteotomy"[Mesh]) OR osteotomy) AND ((((((((((("Tranexamic Acid"[Mesh]) OR Tranexamic Acid) OR AMCHA) OR trans-4-(Aminomethyl) cyclohexane carboxylic Acid OR t-AMCHA) OR AMCA) OR Anvitoff) OR Cyklokapron) OR Ugurol) OR KABI 2161) OR Spotof OR Transamin OR Amchafibrin) OR Exacyl). Randomized controlled trials (RCTs), cohort or case-control studies that compared TXA with placebo or blank for reducing blood loss or hemoglobin decrease in patients undergoing HTO were included. There were no restrictions on language or country of publication. In addition, we searched the relevant articles and reference lists manually to identify other potentially eligible publications.

### Eligibility criteria

Relevant studies were screened by titles and abstracts, and then the eligibility criteria were applied.

Studies that met the PICOS criteria were included as follows: (1) Patients: patients undergoing HTO. (2) Intervention: TXA applied. (3) Comparison: comparing TXA with placebo or blank. (4) Outcomes: the outcomes concerning efficacy included total blood loss, hemoglobin decrease, drain output, and wound complications; the outcomes concerning safety included postoperative thromboembolic complications. The total blood loss was set as the primary outcome. (5) Study design: randomized controlled trials (RCTs), cohort or case-control studies. The exclusion criteria for the studies were as follows: (1) patients suffered from the non-HTO osteotomy. (2) Not comparing TXA with placebo or blank. (3) Outcomes do not include total blood loss.

### Data extraction

Two reviewers independently extracted data from the selected studies and were subsequently cross-checked for any unwanted human-error. Any disagreements between the two reviewers were resolved by their mutual consensus or were further reviewed by the third reviewer. Variables recorded for this study included general information of patients, total blood loss, hemoglobin decrease, drain output, wound complications, and thromboembolic events.

### Quality assessment

Two authors independently assessed the quality of each study by using the Newcastle–Ottawa Scale (NOS). Studies were scored on a 9-star NOS scale, where a score of ≥ 6 stars was considered a high-quality study. The scale included the following items: representativeness of the exposed cohort, selection of the non-exposed cohort, ascertainment of exposure, demonstration that outcome of interest was not present at start of study, assessment of outcome, follow-up period, and follow-up rate.

### Statistical analysis

All statistical analyses were performed using RevMan version 5.3 (The Cochrane Collaboration, Copenhagen, Denmark). The risk ratio (RR) with a 95% confidence interval (CI) was calculated for dichotomous variables, such as wound complications and thromboembolic events. The mean difference (MD) with 95% CI was calculated for continuous variables, such as total blood loss, hemoglobin decrease, and drain output. The heterogeneity was tested depending on the P value and I^2^ using the standard *χ*2 test. I^2^ < 50%, *P* ≥ 0.1, was treated with no significant statistical heterogeneity, a fixed-effects model was employed. Otherwise, a random-effect model was used. And a sensitivity analysis was performed on the impact of each study on the overall outcome estimate by omitting one study per round, in case there were any huge statistical heterogeneities.

## Results

### Search results and quality assessment

**Fig. 1:**
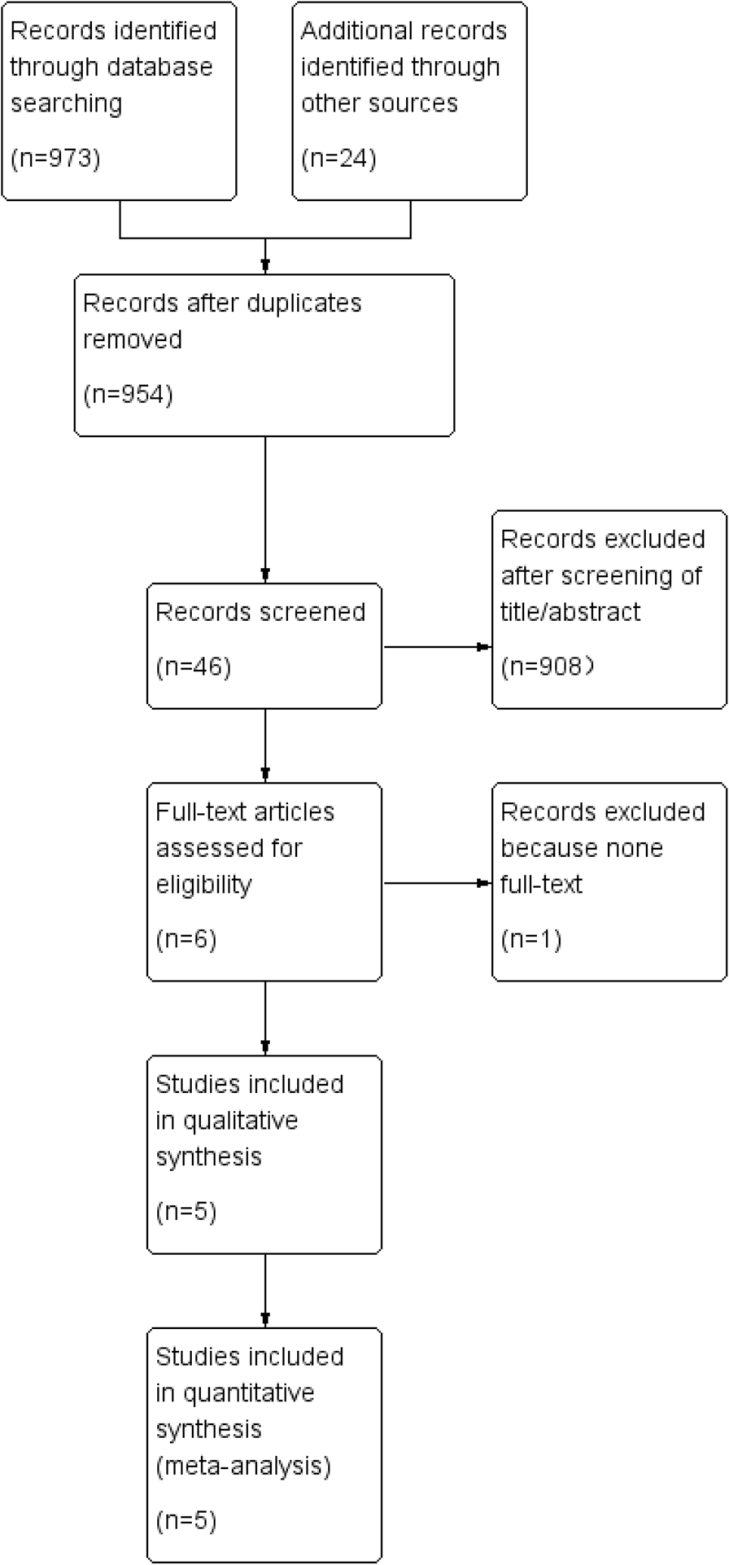
Flow diagram of the meta-analysis

Figure 1 shows the processes of study search, selection, and exclusion. A total of 997 studies were identified after the initial search from the PubMed (MEDLINE), EMBASE, and Cochrane Library databases. A total of 954 unique studies remained eligible for the final study, while the duplicates were removed. Then, 908 studies were excluded based on their titles and/or abstracts, 6 remaining studies were evaluated through full text according to the selection criteria. Finally, 5 studies [6, 19–22] consisting of 532 patients, 258 patients in TXA group, and 274 in the control group were included in this meta-analysis. Of the included studies, one was a prospective comparison study, and the remaining four were retrospective comparison studies. One study reported the use of topical and intravenous TXA [20], one study reported the use of topical TXA only [19], and in other studies, intravenous TXA was administered [6, 21, 22]. The characteristics of the included studies were listed in Table 1. According to the NOS, one study scored 8 stars [22], three studies scored seven stars [6, 19, 21], and one study scored 6 stars [20]. The details were exhibited in Table 2.

**Table 1:** Characteristics of the included studies

Author	Year	Study type	Operation	TXA route	TXA group			Control group		
					Sample	Age	Intervention	Sample	Age	Intervention
Chen	2020	RCS	HTO	IV&Topical	52	58.3 ± 10.4	TXA	48	56.6 ± 10.2	None
Kim	2018	RCS	HTO	IV	75	55.0 ± 6.8	TXA	75	55.7 ± 5.5	None
Ni	2020	PCS	HTO	IV	50	52.5 ± 2.8	TXA	50	52.9 ± 3.1	Normal saline
Palanisamy	2018	RCS	HTO	IV	66	58 ± 5	TXA	86	57 ± 6	None
Suh	2018	RCS	HTO	Topical	15	60 ± 5.6	TXA	15	56 ± 5.7	None

*PCS* prospective comparison study, *RCS* retrospective comparison study, *HTO* high tibial osteotomy, *IV* intravenous, *TXA* tranexamic acid

**Table 2:** Assessment of the quality of the studies based on the Newcastle–Ottawa Scale

Study	Selection (out of 4)				Comparability (out of 2)	Outcomes (out of 3)			Total (out of 9)
	(1)	(2)	(3)	(4)		(5)	(6)	(7)	
Chen	*	*	*	*	*		*		6
Kim	*	*	*	*	*		*	*	7
Ni	*	*	*	*	**		*	*	8
Palanisamy	*	*	*	*	**	*			7
Suh	*	*	*	*	**		*		7

(1) Representativeness of the exposed cohort, (2) selection of the non-exposed cohort, (3) ascertainment of exposure, (4) demonstration that outcome of interest was not present at start of study, (5) assessment of outcome, (6) was follow-up long enough for outcomes to occur, (7) adequacy of follow-up of cohorts

### Meta-analysis results

#### Total blood loss

**Fig. 2:**
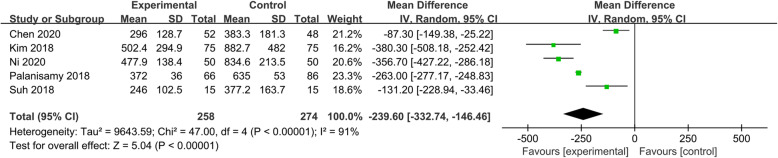
Forest plot showing the total blood loss of patients undergoing HTO between TXA and control groups

All five included studies [6, 19–22] involving 532 patients were evaluated for total blood loss after HTO. The random-effect model was applied for heterogeneity among these studies (*P* < 0.00001, I^2^ = 91%). A significant difference was detected in total blood loss between the two groups (MD = − 239.60, 95% CI − 332.74 to − 146.46, *P* < 0.00001, Fig. 2).

#### Hemoglobin decrease

**Fig. 3:**
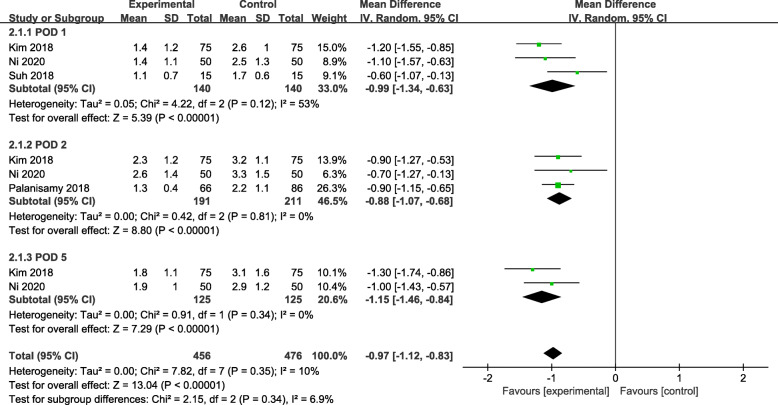
Forest plot showing the hemoglobin decrease of patients undergoing HTO between TXA and control groups

Four studies were reported the changes related to hemoglobin level: three of the studies reported the hemoglobin decrease for the post-operation day (POD) 1 [6, 19, 22], three of the studies reported the hemoglobin decrease for the POD 2 [6, 21, 22], and two of the studies reported data for POD 5 [6, 22]. The heterogeneities among studies were not statistically significant (P = 0.35; I^2^ = 10%). A significant difference was detected in hemoglobin decrease for the POD 1 (MD = − 0.99, 95% CI − 1.34 to − 0.63, *P* < 0.00001), POD 2 (MD = − 0.88, 95% CI − 1.07 to − 0.68, *P* < 0.00001), and POD 5 (MD = − 1.15, 95% CI − 1.46 to − 0.84, *P* < 0.00001) between the two groups (Fig. 3).

#### Drain output

**Fig. 4:**
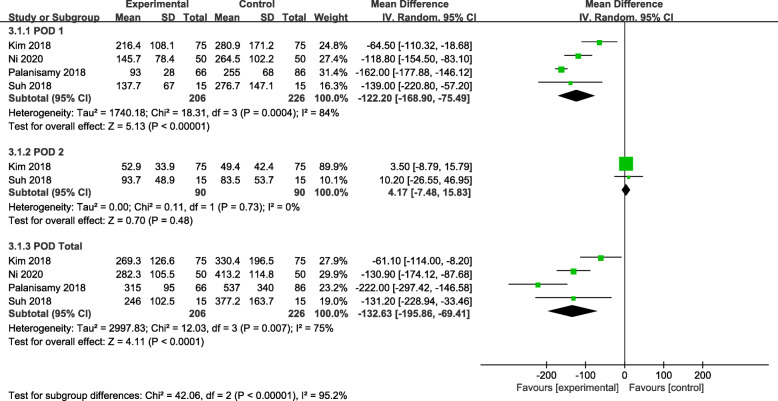
Forest plot showing the drain output of patients undergoing HTO between TXA and control groups

Four studies reported the changes of drain output: four studies reported the drain output for the POD 1 [6, 19, 21, 22], two of the studies reported the drain output for the POD 2 [6, 19], and four studies reported data for total drain [6, 19, 21, 22]. The random-effect model was applied for heterogeneity among these studies (*P* = 0.007, I^2^ = 75%,). A significant difference was detected in drain output for the POD 1 (MD = − 122.20, 95% CI − 168.90 to − 75.49, *P* < 0.00001) and POD total (MD = − 132.63, 95% CI − 195.86 to − 69.41, *P* < 0.00001) between the two groups (Fig. 4).

#### Wound complications

**Fig. 5:**
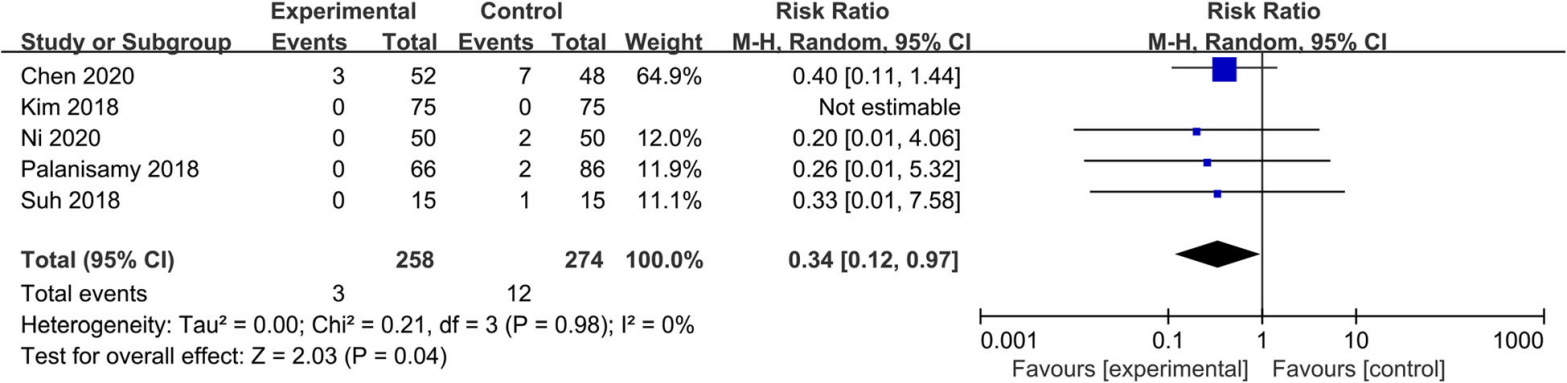
Forest plot showing the wound complications of patients undergoing HTO between TXA and control groups

Five studies [6, 19–22] reported data related to wound complications. The heterogeneities among these studies were not statistically significant (*P* = 0.98, I^2^ = 0%). A significant difference (RR = 0.34, 95% CI 0.12 to 0.97, *P* = 0.04) was detected in wound complications between the two groups (Fig. 5).

#### Thromboembolic events

Four studies provided data for the thromboembolic events: one study [20] showed two patients with thrombotic events in the TAX group and four in the control group. Other studies [6, 21, 22] showed no thrombotic events happened in either group. Non-significant differences (RR = 0.46, 95% CI 0.09 to 2.41, *P* = 0.36) were found in the incidence of thromboembolic events between the study groups.

#### Blood transfusion rate

**Fig. 6:**
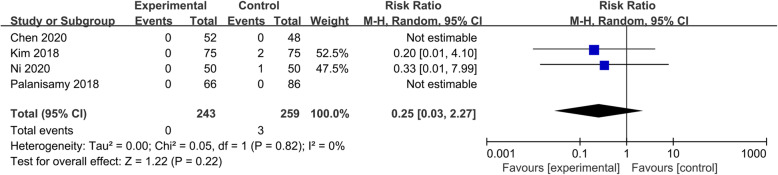
Forest plot showing the blood transfusion rate of patients undergoing HTO between TXA and control groups

Four studies [6, 20–22] reported data for the blood transfusion rate. The heterogeneities among these studies were not statistically significant (*P* = 0.82, I^2^ = 0%). Non-significant differences (RR = 0.25, 95% CI 0.03 to 2.27, *P* = 0.22) were found in the incidence of blood transfusion rate between the two groups (Fig. 6).

## Discussion

### Main findings

To the best of our knowledge, this is the first meta-analysis report to evaluate the efficacy and safety of TXA with respect to appropriate control in patients with only HTO, without involving other osteotomy surgery. Based on the outcomes of pool of 5 studies, the most important finding of this meta-analysis was that the administration of TXA could significantly reduce total blood loss (MD = − 239.60, 95% CI − 332.74 to − 146.46, *P* < 0.00001) and wound complications (RR = 0.34, 95% CI 0.12 to 0.97, *P* = 0.04). This is consistent with the evidence that TXA can also reduce total blood loss in arthroplasty, spine, and other orthopedic surgeries [13, 23, 24]. Indeed, blood loss is inevitable when the medullary cavity is exposed during the tibial osteotomy. Moreover, this surgical procedure can cause the formation of bone gaps due to nonunion of long bones and the inability of associated large soft tissues to properly cover the bony space, thereby increasing the risks of wound hematoma, skin infections, and even compartment syndrome during HTO postoperative period [8]. Thus, it is of utmost importance to evaluate the clinical value of TXA to treat these HTO-associated medical conditions.

Several studies have reported that TXA could significantly reduce the hemoglobin drop and drain output in open wedge HTO [6, 19]. The findings of our meta-analysis were consistent with the previous studies, which revealed that TXA could significantly reduce the total blood loss, hemoglobin decline and drain output after HTO. Our findings also agree with that of Wassilew et al. [25] in the sense that HTO patients treated with TXA have exhibited a significant reduction in the total blood loss, hemoglobin drop, and drain output than those in placebo-treated HTO patients. Although tourniquets are widely used in orthopedic surgery to control perioperative blood loss, there is no consensus on how to use a tourniquet in HTO toward improved outcomes [26–28]. However, most orthopedic surgeons choose to use a tourniquet in HTO because of the increased risk of excessive blood loss due to the opening of the medullary cavity. All the included studies have used tourniquet intraoperatively to reduce blood loss and have reported their corresponding blood transfusion rates. Ni et al. [22] has reported that the need for blood transfusion in one patient in the control group, while Kim et al. [6] has documented that two patients received a blood transfusion in the control group. However, other studies have shown no record of blood transfusion in both TXA and control groups [19–21]. Taken together, these results suggest that there is no obvious evidence that TXA could reduce blood transfusion rate in patients undergoing HTO (RR = 0.25, 95% CI 0.03 to 2.27, *P* = 0.22). The results were not consistent with other research studies that reported that TXA could reduce the risk of transfusion rate in specific orthopedic surgeries [24, 25, 29]. However, Guo et al. [30] reported no statistically significant difference in transfusion rates between the TXA and control groups, which was consistent with our findings. In our opinion, the main reason for these discrepancies could be the different types of surgeries involved in these published studies. Moreover, different hospitals or orthopedic surgeons often follow their own standards of blood transfusion depending on the patient’s necessity.

Wound complications commonly occur in these patients after HTO, and the cumulative rate of all complications was 41.2% [7, 9, 31]. We did find that the effect of TXA was significantly in reducing various wound complications, such as wound hematoma and infection. Although all of the five included studies showed no significant difference between the two groups in this respect, the pooled results suggested that TXA effectively reduced wound complications (RR = 0.34, 95% CI 0.12 to 0.97, *P* = 0.04). Similarly, Xie et al. [32] indicated that TXA could effectively reduce post-operative wound complications in patients with calcaneal fractures (7.3% versus 23.8%; *p* = 0.036). Notably, we could find only one meta-analysis [33] that has evaluated the clinical efficacy of TXA in managing wound complications in patients undergoing osteotomy, and reportedly there is no significant decrease between the TXA and control groups. Further studies with a larger sample size are required to arrive at a statistically significant conclusion on this matter.

Thromboembolic events (including deep vein thrombosis and pulmonary embolism) are considered fatal complications caused by orthopedic surgery and may even lead to death [34, 35]. Four studies were included reporting data for thromboembolic events. Amongst them, only one study [20] showed two patients with thrombotic events in the TAX group and four in the control group. But this event was not observed between the two study groups of patients in other previously published studies. The current meta-analysis implies that TXA would not significantly increase the incidence of thromboembolic events (RR = 0.46, 95% CI 0.09 to 2.41, *P* = 0.36) in patients undergoing HTO, which is in agreements with previous studies [36, 37]. Notably, the administration of TXA has been found to be safe and well-tolerated in optimum dose, which inhibits fibrinolysis only in the wound, but not in the general circulation system [38]. In this regard, Stowers et al. [39] and Yuan et al. [40] also consistently reported that TXA did not increase the risk of thromboembolic events in patients undergoing total knee arthroplasty.

In addition, the optimal dose and administration method of TXA in HTO patients are still controversial. The clinical application of TXA involves various methods. Amongst them, intravenous and topical administrations of TXA are the most widely used methods in orthopedic surgery [41]. Palanisamy et al. suggest that 2 g of TXA were intravenously infused before tourniquet application in HTO, and the same dose of TXA was repeated in 3 h [21]. While Ni et al. recommend that patients should receive TXA intravenously at a dose of 50 mg/kg 10 min before the tourniquet deflation [22]. Differently, Suh et al. has pointed out that 2 g of TXA in 20 mL saline can be topically administered at the osteotomy site [19]. However, Li et al. [42] have revealed that intravenous combined with topical TXA administration has no additional efficacy compared with intravenous administration alone in HTO. In joint-related surgery, such as total knee arthroplasty (TKA), there is a number of studies comparing the efficacy of intravenous versus topical administration methods [43, 44]. Studies have shown that the combined administration of intravenous and topical TXA in primary TKA may be more effective [45, 46]. But in the case of HTO, it is an extra-articular procedure. Although topical TXA can increase the local concentration of TXA and can act more effectively to reduce blood loss [47]; however, it is important to note that the bone gap generated by HTO is not an airtight space, and the topically applied TXA may penetrate into the soft tissue space causing swelling and further worsening the wound complications. Therefore, intravenous TXA administration might be a better method for HTO patients, but more large-scale studies are needed to prove it. Notably, intravenous TXA administration includes single-dose, multiple-dose, and continuous infusion methods. Interestingly, one study has reported that there were not any significant beneficial effects of three doses of TXA in TKA as compared to a single-dose treatment method [48]. However, Lei et al. [49] have suggested that the 5-dose intravenous TXA can further decrease any hidden blood loss as well as the maximum hemoglobin drop in total hip arthroplasty. This difference might be due to the setting of multiple-dose and the type of operation performed in that particular case. And another study has demonstrated that a single dose of TXA at 30 mg/kg body-weight is as effective as a continuous infusion in patients undergoing TXA treatment [50].

## Limitations

Several limitations of this study also warrant consideration. First, only five studies were included in the final analysis. Although the outcomes of total blood loss, hemoglobin decrease, drain output, wound complications, thrombotic events, and blood transfusion rate were reportedly available, full-text published articles about TXA administration in patients undergoing HTO are relatively rare. Second, only one study was RCT, and others were non-RCT cohort studies resulting in an uncontrolled bias. Third, significant heterogeneity was observed in the outcomes for total blood loss. This might have resulted because the mean total blood loss was estimated by different strategies. It would be better for future studies to follow the same method for total blood loss calculation.

## Conclusions

This meta-analysis of the available evidence implies that TXA reduces total blood loss, hemoglobin decrease, drain output, and wound complications without increasing the incidence of thromboembolic events in patients undergoing HTO. But there is no obvious evidence that TXA could reduce the blood transfusion rate in these patients. More large-scale and well-designed RCTs are required to verify the efficacy and safety of TXA in patients undergoing HTO.

## Data Availability

All data generated or analyzed during this study are included in this published article and its supplementary information files.

## Abbreviations

TXA: Tranexamic acid
HTO: High tibial osteotomy
PRISMA: Preferred Reporting Items for Systematic Reviews and Meta-Analyses
NOS: Newcastle–Ottawa Scale
RCTs: Randomized controlled trials
RR: Risk ratio
CI: Confidence interval
MD: Mean difference
TKA: Total knee arthroplasty
